# Physician burnout, associated factors, and their effects on work performance throughout first-year internships during the COVID-19 pandemic in Thailand: a cross-sectional study

**DOI:** 10.1186/s12889-025-23172-7

**Published:** 2025-05-28

**Authors:** Vithawat Surawattanasakul, Penprapa Siviroj, Wuttipat Kiratipaisarl, Wachiranun Sirikul, Vitchayut Phetsayanavin, Chantarateera Pholvivat, Natcha Auernaruemonsuk, Chanon Lamlert

**Affiliations:** 1 Department of Community Medicine, Faculty of Medicine, Chiang Mai University, Chiang Mai, Thailand; 2 Environmental Medicine and Occupational Medicine Excellent Center, Faculty of Medicine, Chiang Mai University, Chiang Mai, Thailand; 3 Department of Psychiatry, Faculty of Medicine, Chulalongkorn University, Bangkok, Thailand; 4 Department of Community, Family and Occupational Medicine, Faculty of Medicine, Khon Kaen University, Khon Kaen, Thailand; 5 Department of Psychiatry, Faculty of Medicine, Ramathibodi Hospital, Mahidol University, Bangkok, Thailand; 6 Department of Surgery, Ratchaphiphat Hospital, Bangkok, Thailand

**Keywords:** Burnout, Professional satisfaction, Colleague support, Work performance, Intern physicians

## Abstract

**Background:**

Burnout has become a significant occupational concern for physicians who have recently graduated, attributed to their prolonged exposure to workplace stressors, poor work-life balance, and limited patient care experience. These challenges posed during the COVID-19 pandemic, placed unprecedented stress on healthcare systems and first-year interns navigating their careers. This study aimed to assess the prevalence of burnout among first-year intern physicians (1st IPs), investigate the factors contributing to burnout, and explore potential correlations between burnout and work performance.

**Methods:**

This cross-sectional study was carried out between June and July 2022, enrolling 412 1st IPs in Thailand. These participants completed a questionnaire through an online web-based platform. The questionnaire included the Maslach Burnout Inventory-Human Services Survey for Medical Personnel to assess burnout, as well as items addressing factors related to working conditions. These factors included colleague support, academic counselling, professional satisfaction, income and workload balance, medical errors, work performance, resignation thoughts, and suicidal ideation. Data were analysed using multivariable logistic regression.

**Results:**

Among the participants, 58.5% were female, with an average age of 25.59 years (SD 2.18). A significant proportion (81.2%) worked more than 80 h per week. Nearly half, 48.1% experienced burnout, characterized by high levels of emotional exhaustion (83.5%), depersonalization (74.8%), and low personal accomplishment (66.5%). In the adjusted model, physicians lacking support from their colleagues had higher levels of burnout (adjusted odds ratio [aOR] 2.56, 95% CI 1.18 to 5.58). Those dissatisfied with their professional life were more likely to experience burnout compared to those who were satisfied (aOR 4.52, 95% CI 2.31 to 8.84). Burnout was also significantly associated with poor work performance (aOR 2.14, 95% CI 1.08 to 4.21), while no association was found between burnout and suicidal ideation.

**Conclusions:**

Our findings revealed a significantly high prevalence of burnout among 1st IPs in Thailand. This burnout was associated with inadequate colleague support and professional dissatisfaction, ultimately resulting in poor work performance. To address these issues, mentorship programs and buddy support systems, along with adherence to recommended work-hour guidelines, are crucial to mitigate burnout and improve work performance.

**Supplementary Information:**

The online version contains supplementary material available at 10.1186/s12889-025-23172-7.

## Background

Burnout is an occupational phenomenon formally recognized by the World Health Organization (WHO) in the International Classification of Diseases, 11th Revision (ICD-11), under code QD85. It is defined as a syndrome that arises from chronic workplace stress that has not been adequately managed. This condition is characterized by three key symptoms: feelings of energy depletion, mental distancing from work, and reduced professional efficacy [1, 2]. The Maslach Burnout Inventory (MBI) remains the most extensively utilized tool for assessing burnout, identifying three dimensions: emotional exhaustion (EE), depersonalization (DP), and personal accomplishment (PA) [3]. Burnout is a significant concern within the medical profession, with serious consequences for healthcare providers, patients, and healthcare systems. For healthcare professionals, it adversely affects physical, psychological, and social well-being, manifesting as early career attribution, depression, and even suicidal ideation. Among patients, physician burnout is associated with a higher incidence of harmful clinical errors, which can result in serious harm and poor quality of care. In the healthcare systems, burnout leads to decline in the overall efficiency of the system [4–6].

Physician burnout is a crucial concern, predominantly stemming from the demanding and stressful nature of their work environments. Before the COVID-19 pandemic, global burnout prevalence among physicians, as assessed using the MBI, was reported 67%, with high EE at 72.0%, high DP at 68% and low PA at 63.2% [7]. In comparison, global burnout rates among medical residents were lower, with overall burnout documented at 35.7%, high EE at 38.9%, high DP at 43.6%, and low PA at 34.5% [8]. In Thailand, MBI-based studies among residents showed variable levels of burnout, with high EE ranging from 20.7 to 43.7%, high DP from 35.1 to 52.8%, and low PA between 0% and 48.6% [9, 10]. Similar trends were observed among medical interns, where studies in Ireland reported overall burnout rates ranging from 69.5 to 72.6%, with 43.5% of interns experiencing psychological distress [11, 12]. Previous studies emphasize that physicians in their initial year of clinical practice experience particularly high levels of stress and burnout [13, 14]. During the COVID-19 pandemic, systematic reviews documented a global increase in burnout among healthcare professionals with overall burnout rates among physicians and nurses reaching 66% (95% CI 51–81%) [15, 16]. In Thailand, burnout rates among residents during the pandemic was reported at 46.3%, with high EE ranging from 50.7 to 57.1%, high DP from 36.1 to 38.8%, and low PA from 51.4 to 94.0% [17, 18].

Physician burnout usually arises from prolonged exposure to excessive work-related stressors, influenced by multiple factors that can be divided into individual and workplace-related factors. Individual factors include personal attributes and challenges, such as gender differences [19–21], financial stress [22, 23], professional satisfaction [24, 25], and insufficient sleep [18, 26]. Additionally, personal challenges, such as thoughts of resignation [18, 27], low self-efficacy, poor work-life balance, and perceived poor mental health [23], further increase vulnerability to burnout. Workplace-related factors, on the other hand, stem from organizational and systemic issues. These include lengthy training programs [24], excessive workloads [24, 28], and irregular schedules [24], all of which contribute to fatigue and disrupt work-life balance. Furthermore, the lack of access to counselling services [18], inadequate support from colleagues [24, 25, 29], and insufficient mental health resources [22], exacerbate work-related stress and feelings of exhaustion.

In Thailand, newly graduated physicians embark on a three-year program following six years of education [30], with the first year as intern physicians posing significant challenges. This phase involves adapting to new work environments, increased responsibilities, and direct patient care, all of which heighten burnout risks, particularly during the COVID-19 pandemic. We hypothesized that medical interns are susceptible to burnout syndrome during the COVID-19 pandemic [20, 31], sharing similar risk factors with their colleagues in the medical profession. These risk factors encompass prolonged exposure to a demanding workload, irregular work hours, and the psychological stress stemming from their limited experience in patient care. Additionally, the mandatory one-year placement in government hospitals, often assigned randomly [32], creates unpredictable and challenging conditions. This placement holds significant importance during the initial year following graduation, as successful completion is a prerequisite for advancement into any resident training program. Consequently, the wellbeing of intern physicians has received limited attention due to the unpredictable, challenging, and mandatory nature of their working conditions. Moreover, the lack of nationwide data on burnout and its associated risk factors among intern physicians in Thailand further underscores the importance of focused investigation. Therefore, this study aimed to investigate the prevalence of burnout, its associated factors, and its potential correlations with professional performance among first-year intern physicians (1st IPs) in Thailand.

## Methods

### Study population

This national cross − sectional study focused on recruiting individuals who were required to work as 1st IPs in Thailand. The study employed a web-based online questionnaire which was available for completion from 1st June 2022 to 30th July 2022, coinciding with the final two months of the internship in the academic year. The questionnaire was distributed using web-based tools accessible across the country. To invite participants, study posters were posted on internet websites, specific instant messaging channels were used for intern physicians (for example a Line application), and media platforms like Facebook. The web-based online questionnaire was interactive in design, and burnout scores were reported and interpreted at the end of the test based on the responses of participants. The questionnaire items are available in Supplementary file 1. Strict emphasis was placed on the maintenance participant anonymity, and no identifiable information was requested.

**Fig. 1:**
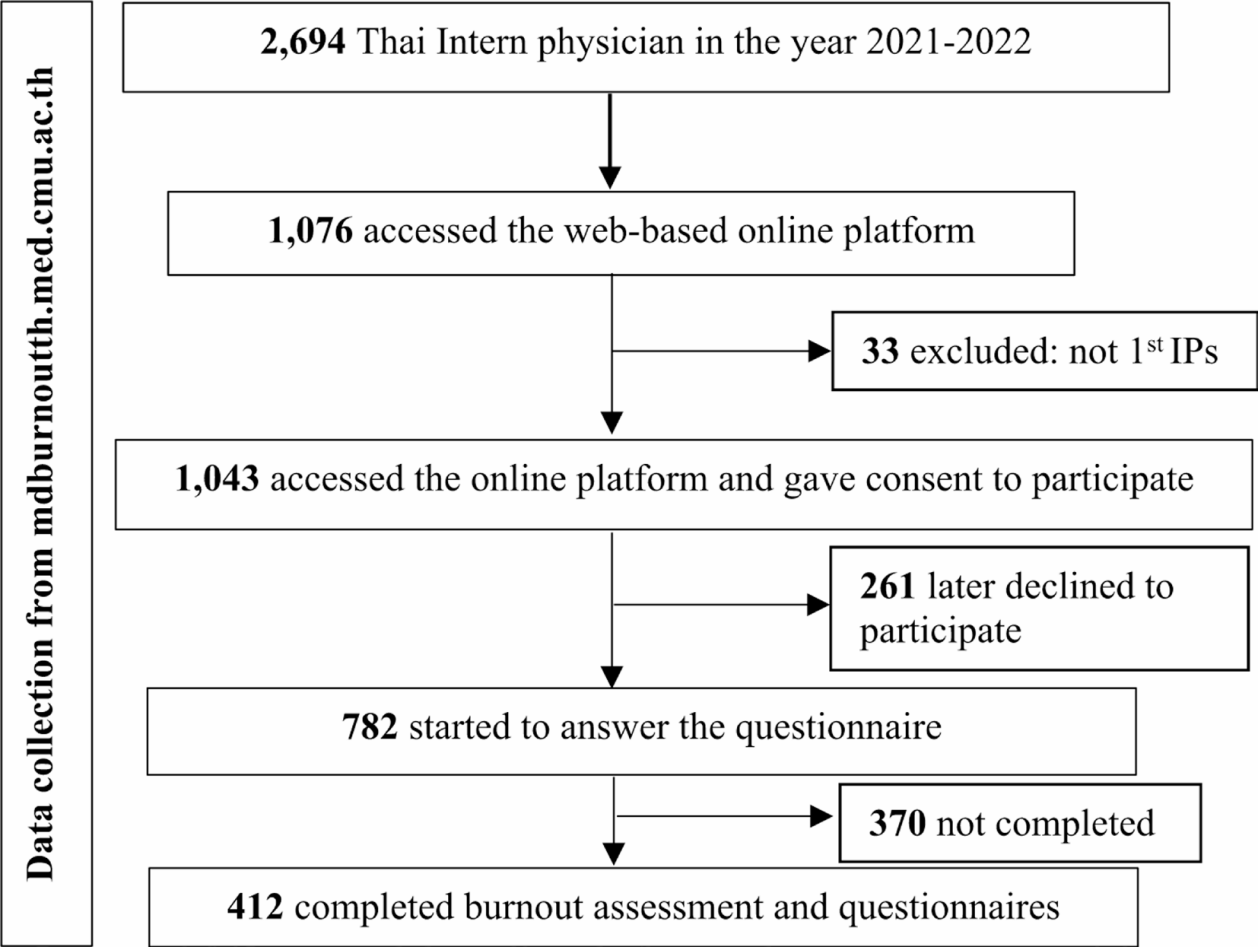
Flow diagram showing the recruitment of Thai intern physicians in this study (*n* = 412)

According to the Medical Council of Thailand, there were 2,694 intern physicians working in the academic year 2021. The sample size for this study was calculated using EpiInfo™ version 7.2 [33], taking into account an expected frequency of 47.4% [34], a confidence level of 97%, an acceptable margin of error of 5%, and a design effect of 1.0. Based on these calculations, the total sample size was determined to be 400. For the survey, 1,076 participants accessed the online platform to complete the survey. However, 33 participants were excluded because they were not physicians, and an additional 261 participants did not provide their consent to participate in the study. 782 participants started filling in the questionnaire. Finally, 412 participants, completed the burnout assessment (Fig. 1). Before data collection, all participants signed informed consent forms, which were approved by the Institutional Review Board at the Faculty of Medicine, Chiang Mai University, Thailand (Reference number: 079/2022).

Figure 1 Flow diagram showing the recruitment of Thai intern physicians in this study (*n* = 412).

### Data collection

Burnout was assessed using the Maslach Burnout Inventory-Human Services Survey for Medical Personnel, MBI-HSS (MP), purchased from Mind Garden, Inc., CA, USA. This inventory consisted of 22 items that had been translated into Thai and used in previous studies [9, 10, 18]. Participants rated each item on a 7-point Likert scale, ranging from 0 to 6, based on the frequency of occurrence. The rating scale for the EE and PA subscales was as follows: “0 = Never, 1 = A few times a year or less, 2 = Once a month or less, 3 = A few times a month, 4 = Once a week, 5 = A few times a week, and 6 = Every day”. It is important to note that the PA subscale had an inverse rating score. In this study, the Cronbach’s alpha values for the subscales were as follows: EE subscale = 0.926, DP subscale = 0.811, and PA subscale = 0.833. Burnout was characterized by high scores of the EE and DP subscales, as well as low scores of the PA subscale. The classification of burnout and the determination of subscale cut points were based on the suggestions provided in the MBI manual, third edition [3]. EE levels were categorized into low (0–18), moderate (19–26), and high (≥ 27). DP levels were classified into low (0–5), moderate (6–9), and high (≥ 10). PA levels were defined as high (≥ 40), moderate (34–39), and low (0–33).

The working conditions of physicians were assessed through a series of questions focusing on various aspects. These included: (1) The frequency of support from colleagues (not/ sometimes/ most of time); (2) The frequently of academic counselling provided by colleagues (not/ sometimes/ most of time); (3) The balance between income and workload (balance/imbalance); (4) Professional satisfaction (satisfied/ dissatisfied); and (5) Your recent emotional state regarding unhappiness and depression (strongly disagree/ disagree/ neutral/ agree/ strongly agree).

The work performance of physicians was assessed by posing specific questions about their performance after one year of working in the medical profession. The assessment included the following aspects: (1) Thoughts of resignation during the internship: participants were asked if they had contemplated resigning during their internship and whether they eventually resigned within a year of starting work. The response options for this question were “yes” or “no.”; (2) Self-reported medical errors: Participants were requested to self-report any clinically harmful errors, clinically harmless errors, wrong investigations, or wrong prescriptions they had made in the past three months. The response options for this question were “yes” or “no.”; (3) Self-assessment of performance: Participants assessed their own medical practice performance on a scale ranging from 0 to 10. Scores below 7 on this scale were considered indicative of poor performance; and (4) Suicidal ideation: Participants were asked as to whether they had contemplated attempting suicide while at work during their internship. The response options for this question were “yes” or “no.”

### Statistical analysis

All statistical analyses were performed using the STATA software package (Stata Corp. 2019. Stata Statistical Software: Release 16, College Station, TX, USA: Stata Corp LLC.). Survey data collected from the web-based platform was exported for analysis. Descriptive statistics, including frequency with percentages for categorical variables and mean values with standard deviation for continuous variables, were used to summarize the data, depending on the parametric nature of the variables. The association between working conditions and burnout, as well as burnout and performance, was examined using binary logistic regression. To explore the association between the burnout and contributing factors, multivariable logistic regression was employed with adjustments made for potential confounders, including sex, age, hospital affiliation, regional location of work, sleep duration hours, and total working hours. Furthermore, the association between burnout and work performance was analysed, flowing adjustment for potential confounders such as sex, age, hospital affiliation, regional location of work, sleep duration hours, total working hours, and feelings of unhappiness and depression. All statistical analyses were conducted using a two-tailed test, and a significance level of less than 0.05 was considered significant. The findings of the study were reported following the guidelines outlined in STROBE (Strengthening the reporting of observational studies in Epidemiology) [35].

## Results

### Characteristics, working conditions and work performance

Data from the questionnaires completed by 412 intern physicians were analysed, resulting in a response rate of 15.3% (412/2694). Table 1 presents the characteristics, working conditions, and performance of the participants. The intern physician had a mean ± SD age of 25.59 ± 2.18 years. On average, they slept for 5.58 ± 1.12 h per day. Notably, the participants worked an average of 69.27 ± 22.70 h per week, and 81.2% of them worked over 80 h per week. Among the participants, 17.0% received frequent support from their colleagues, and 16% received frequent academic counselling. The majority of intern physicians (80.7%) perceived an imbalance between their income and workload, while 31.5% expressed professional dissatisfaction. Regarding work performance, 40.4% of participants reported making clinically harmful errors. Additionally, the majority of participants rated their performance as poor (68.2%). During their internship, a notable proportion of participants (78.6%) considered resigning from their jobs. Notably, 15.7% of intern physicians reported experiencing suicidal ideation. The province-by-province distribution of survey respondents from all regions of Thailand is shown in Supplementary file 2.

**Table 1:** Characteristics, working conditions, and performance of intern physicians (*n* = 412)

Characteristics, working conditions, and performance	*n* (%), Mean (SD)
**Characteristics** (*n* = 412)	
Sex	
Female	241 (58.5)
Male	171 (41.5)
Age (years)	25.59 (2.18)
Marital status, Single	407 (98.8)
Income per month (USD)	1829.99 (492.51)
Had underlying disease	175 (42.5)
Taking medication	87 (21.1)
**Daily sleep duration**	
Sleep duration (hours)	5.58 (1.12)
**Weekly working hours (*n*** **= 314)**	
Total working hours, min-max	69.27 (22.70), 44–168
Working hours ≥ 80 h	255 (81.2)
**Working conditions (*n*** **= 327)**	
**Support from colleagues**	
Not and sometimes	257 (62.4)
Most of time	70 (17.0)
**Academic counselling service**	
Not and sometimes	261 (63.3)
Most of the time	66 (16.0)
**Imbalance of income and workload**	264 (80.7)
**Professional satisfaction**	
Satisfied	224 (68.5)
Unsatisfied	103 (31.5)
**Unhappy and depressed feeling (*n*** **= 225)**	
Agree to strongly agree	176/225 (78.2)
Neutral to strongly disagree	49/225 (21.8)
**Self-reported medical errors in the past 3 months (*n*** **= 245)**	
Clinically harmful error	99/245 (40.4)
Clinically harmless error	110/245 (44.9)
Wrong investigation	149/245 (60.8)
Wrong prescription	132/245 (53.9)
**Working performance**	
Self-assessment of poor performance (*n* = 255)	174/255 (68.2)
Thoughts of resignation during the internship (*n* = 327)	257/327 (78.6)
Resigned within one year of work (*n* = 412)	49/412 (11.9)
Suicidal ideation (*n* = 255)	40/255 (15.7)

Abbreviations: SD, Standard deviation; USD, US Dollars, 1 USD = 36.36 Thai Baht

### Prevalence of burnout and its subscales

**Fig. 2:**
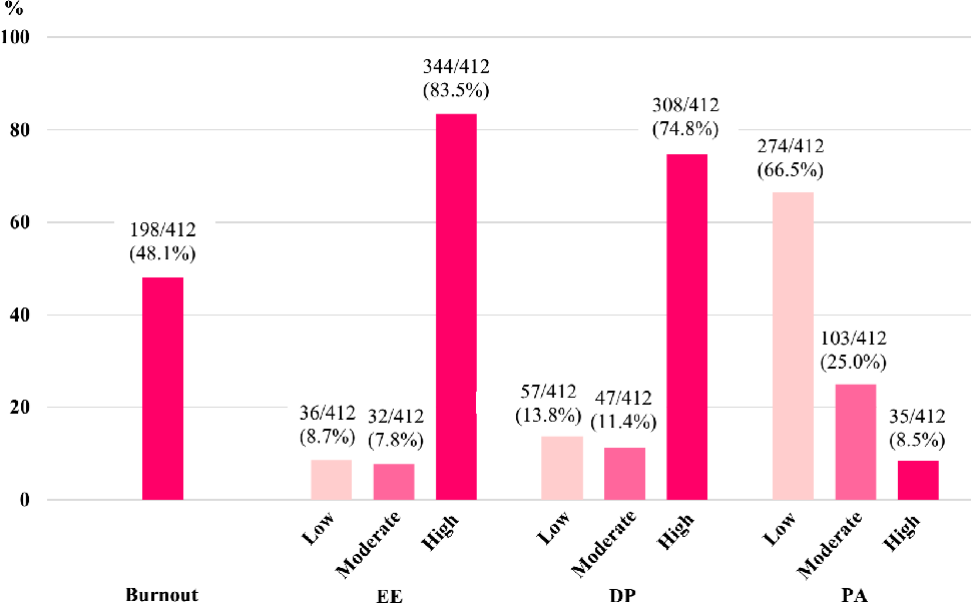
Bar Plots Illustrating the Prevalence of Intern Physician Burnout and Subscales by Degree of Burnout (*n* = 412). Abbreviations: EE, emotional exhaustion; DP, depersonalize; PA, personal accomplishment

Figure 2 shows that 48.1% of intern physicians had experienced burnout. The burnout subscales revealed high levels of EE (83.5%), DP at 74.8%, and low levels of PA at 66.5%. The percentage of low and moderate levels of EE were 8.7% and 7.8% respectively, while the percentage of low and moderate levels of DP were 13.8% and 11.4% respectively. The percentage of high and moderate levels of PA were 8.5% and 25.0% respectively. The means of the burnout questions for each scale, as indicated in the forest plot, ranged from 3.53 to 4.68 for EE, 1.73 to 3.53 for DP, and 1.47 to 3.59 for PA (Supplementary file 3).

### Association between working conditions and burnout

Intern physicians who lacked support from their colleagues were more likely to experience burnout (adjusted odds ratio [OR] = 2.56, 95% confidence interval [CI] 1.18 to 5.58). In terms of the subscales, participants who perceived an imbalance between income and workload had a greater likelihood of high EE compared to those who perceived a balance (adjusted OR = 3.40, 95% CI 1.32 to 8.74). Similar, participants who did not receive support from colleagues had higher odds of high DP than those who did (adjusted OR = 2.92, 95% CI 1.37 to 3.22). Furthermore, those who did not receive academic counselling had higher odds of low PA compared to those who did (adjusted OR = 2.11, 95% CI 1.02 to 4.37). Additionally, participants with dissatisfactory professional experiences had higher odds of burnout in all subscales compared to those with satisfactory professional experiences (Burnout, adjusted OR = 4.52, 95% CI 2.31 to 8.84; High EE, adjusted OR = 8.64, 95% CI 2.05 to 36.48; High DP, adjusted OR = 3.72, 95% CI = 1.62 to 8.56; Low PA, adjusted OR = 2.03, 95% CI = 1.04 to 3.94) (see Table 2).

**Table 2:** Binary logistic regression and multivariable logistic regression analysis of the association between working conditions and burnout and subscales among intern physicians (*n* = 412)

Working Conditions	Burnout				High EE				High DP				Low PA			
	cOR (95%CI)	*p* ^b^	aOR^a^ (95%CI)	*p* ^b^	cOR (95%CI)	*p* ^b^	aOR^a^ (95%CI)	*p* ^b^	cOR (95%CI)	*p* ^b^	aOR^a^ (95%CI)	*p* ^b^	cOR (95%CI)	*p* ^b^	aOR^a^ (95%CI)	*p* ^b^
**Support from colleagues**																
Not and sometimes	1.64 (0.96, 2.79)	0.07	2.56 (1.18, 5.58)	0.02	2.35 (1.23, 4.49)	0.01	1.74 (0.69, 4.36)	0.24	1.84 (1.07, 3.14)	0.03	2.92 (1.37, 3.22)	0.01	0.80 (0.32, 2.02)	0.63	1.41 (0.70, 2.85)	0.33
Most of time	1		1		1		1		1		1		1		1	
**Academic counseling service**																
Not and sometimes	1.85 (0.77, 4.47)	0.17	1.22 (0.56, 2.66)	0.62	NA	NA	0.62 (0.22, 1.79)	0.38	0.82 (0.32, 2.07)	0.67	0.79 (0.34, 1.79)	0.57	4.82 (1.81, 12.85)	0.01	2.11 (1.02, 4.37)	0.04
Most of time	1		1		NA	NA	1		1		1		1		1	
**Income and workload balance**																
Imbalance	2.75 (1.53, 4.93)	0.001	1.55 (0.68, 3.49)	0.29	6.38, (3.33, 12.20)	< 0.001	3.40 (1.32, 8.74)	0.01	2.42 (1.37, 4.27)	0.01	1.42 (0.64, 3.15)	0.39	2.03 (0.91, 4.52)	0.08	1.77 (0.84, 3.75)	0.13
Balance	1		1		1		1		1		1		1		1	
**Professional satisfaction**																
Unsatisfied	2.45 (1.49, 4.02)	< 0.001	4.52 (2.31, 8.84)	< 0.001	9.09 (2.76, 29.94)	< 0.001	8.64 (2.05, 36.48)	0.01	4.90 (2.67, 9.00)	< 0.001	3.72 (1.62, 8.56)	0.01	0.29 (0.14, 0.61)	0.001	2.03 (1.04, 3.94)	0.04
Satisfied	1		1		1		1		1		1		1		1	

Abbreviations: cOR, crude odds ratio; aOR, adjusted odds ratio; EE, emotional exhaustion; DP, depersonalize; PA, personal accomplishment; CI, confidence interval

Significant association at odds ratio values were analyzed using binary logistic regression and multivariable logistic regression

^a^ Association adjusted for sex, age, hospital affiliation, regional location of work, total working hours, and sleep duration hours

^b^ The *P* value for each category vs. the reference

NA = All participants reported ‘Not and sometimes’ in ‘Academic counseling service’ were evaluated as high EE

Table 2 Binary logistic regression and multivariable logistic regression analysis of the association between working conditions and burnout and subscales among intern physicians (*n* = 412).

### Association between burnout, medical errors, work performance, thoughts of resignation and suicidal ideation

The participants who experienced burnout were more likely to perceive their performance as poor (adjusted OR = 2.136, 95% CI 1.082 to 4.213). Specifically, those with a high level of EE subscale had higher odds of thoughts of resignation during their internship (adjusted OR = 4.364, 95% CI 1.662 to 11.458) compared to those without burnout. Furthermore, a high level of DP subscale was associated with a higher odds ratio of making clinically harmful errors (adjusted OR = 2.211, 95% CI 1.015 to 4.819) and having poor performance (adjusted OR = 2.119, 95% CI 1.082 to 4.152) compared to those without burnout. In contrast, a low level of PA was associated with higher odds of perceiving poor performance (adjusted OR = 2.535, 95% CI 1.325 to 4.847) compared with those without burnout. There was no association between burnout and all subscales, and suicidal ideation (see Table 3).

**Table 3:** Binary logistic regression and multivariable logistic regression analysis of associations of burnout and subscales with clinical harmful errors, poor performance, thoughts of resignation, and suicidal ideation among intern physicians (*n* = 412)

Burnout	Clinical harmful error				Poor performance				Thoughts of resignation				Suicidal ideation			
	**cOR** **(95%CI)**	***p*** ^**b**^	**aOR** ^**a**^ **(95%CI)**	***p*** ^**b**^	**cOR** **(95%CI)**	***p*** ^**b**^	**aOR** ^**a**^ **(95%CI)**	***p*** ^**b**^	**cOR** **(95%CI)**	***p*** ^**b**^	**aOR** ^**a**^ **(95%CI)**	***p*** ^**b**^	cOR (95%CI)	*p* ^b^	aOR^a^ (95%CI)	*p* ^b^
Overall Burnout	1.76, (1.05, 2.96)	0.03	1.03, (0.49, 2.18)	0.93	2.17, (1.27, 3.71)	0.01	2.14, (1.08, 4.21)	0.03	1.43, (0.72, 2.84)	0.31	1.37, (0.61, 3.10)	0.44	2.57, (1.49, 4.44)	0.001	1.19, (0.52, 2.72)	0.68
	1		1		1		1		1		1		1		1	
High EE	1.50, (0.73, 3.08)	0.27	1.11, (0.39, 3.12)	0.84	3.44, (1.73, 6.83)	< 0.001	2.35, 0.98, 5.64	0.06	6.78, (3.57, 12.89)	< 0.001	4.36, (1.66, 11.46)	0.01	1.87, (0.63, 5.57)	0.26	0.92, (0.25, 3.36)	0.90
	1		1		1		1		1		1		1		1	
High DP	1.991, 1.158–3.423	0.01	2.21, (1.01, 4.82)	0.05	2.64, 1.54, 4.55	< 0.001	2.12, (1.08, 4.15)	0.03	2.68, (1.56, 4.59)	< 0.001	1.47, (0.67, 3.22)	0.34	1.79, (0.85, 3.78)	0.12	2.23, (0.75, 6.61)	0.15
	1		1		1		1		1		1		1		1	
Low PA	0.85, (0.37, 1.95)	0.70	0.77, (0.35, 1.68)	0.51	0.27, (0.08, 0.91)	0.04	2.53, (1.32, 4.85)	0.01	0.80, (0.32, 2.02)	0.63	0.94, (0.43, 2.07)	0.88	0.22, (0.09, 0.55)	0.001	1.24, (0.51, 3.03)	0.63
	1		1		1		1		1		1		1		1	

Abbreviations: cOR, crude odds ratio; aOR, adjusted odds ratio; EE, emotional exhaustion; DP, depersonalize; PA, personal accomplishment; CI, confidence interval

References include burnout vs. no burnout, high vs. low and moderate EE; high vs. low and moderate DP; low vs. high and moderate PA

Significant association between low to moderate/high levels of burnout and its subscales and the impact on working performance at odds ratio values were analyzed using binary logistic regression and multivariable logistic regression

^a^ Association adjusted for sex, age, hospital affiliation, regional location of work, total working hours, sleep duration hours, and feelings of unhappiness and depression

^b^ The *P* value for each category vs. the reference

Table 3 Binary logistic regression and multivariable logistic regression analysis of associations between burnout and subscales with clinical harmful errors, poor performance, thoughts of resignation, and suicidal ideation among intern physicians (*n* = 412).

## Discussion

This first nationwide study investigates the prevalence of burnout among intern physicians in Thailand during their internship across the COVID-19 pandemic in Thailand. The findings reveal a significant association between burnout and various working conditions, including inadequate support from colleagues, limited access to academic counselling services, an imbalance between income and workload, and professional dissatisfaction. Additionally, the results indicate the potential consequences of burnout, such as reduced performance and an increased thought of resignation.

The prevalence rate of burnout among 1st IPs in this study in this study (48.1%) was lower compared to the 69.5–72.5% reported among Irish junior physicians in their first-year internship before the COVID-19 pandemic [11, 12]. This rate was higher rate than the global prevalence of 35.7% among residents as identified in a systematic review before the pandemic [8]. However, the subscales prevalence rates in this study were higher, with 83.5% for EE, 74.8% for DP, and 65% for low PA. These rates exceeded those reported in Irish studies for junior physicians, surpassing the rates reported conducted among physicians before [9, 10] and during the COVID-19 pandemic [17, 18] that encompassed a broader range of Thai resident physicians. Therefore, this study emphasizes the need to interpret the prevalence cautiously, as the high levels observed may be attributed to factors such as the unique challenges of medical internships, participant demographics, and the study’s timing during the COVID-19 pandemic [36]. Increased workloads, patient care difficulties, and pandemic-related pressures likely contributed to heightened anxiety and burnout among intern physicians. The findings highlight the importance of proactive mental health surveillance, Employee Assistance Programs (EAP), regular assessments, personalized communication, and targeted interventions to support at-risk individuals and enhance their ability to manage professional responsibilities effectively [37].

Several factors have been identified as correlating with burnout and its subscales among 1st IPs. Notably, professional satisfaction demonstrated a negative association with burnout across all three subscales. High levels of dissatisfaction were linked to increased burnout, including high EE and DP, and low PA subscales, aligning with previous studies among medical residents [24, 25]. The literature suggests that challenging learning environment and demands of full-time hospital practice contribute to negative professional experiences and dissatisfaction among intern physicians. These challenges arise from a steep learning curve, high-stress levels and the need for continuous adjustment [38, 39]. Furthermore, professional dissatisfaction among 1st IPs may be exacerbated by perceptions of unfairness and injustice in the workplace [39]. Contributing factors include hierarchical structures within the hospital organizations and the inequitable allocation of financial resources, particularly as intern physicians are often required to work extensive hours for salaries that are disproportionately low relative to the demand of their roles [40, 41].

Our findings reveal a significant imbalance between income and workload, contributing to increased levels of EE. While only a minority of participants reported inadequate income, the hourly income rate remains low relative to the substantial workload. This observation aligns with a previous study, which found that Irish junior physicians experienced financial worries and highlighted a significant association between financial stress and burnout [11]. These findings suggest that the incongruity between income and workload may be a contributing factor to burnout [23, 28]. Alarmingly, 80% of participants exceeded the total recommended work hours established by the Accreditation Council for Graduate Medical Education (ACGME), which advises a weekly limit of 80 h [42]. Furthermore, the number of overnight duty hours also surpassed the threshold of 40 h per week established by the Medical Council of Thailand in 2022 [43].

Moreover, our findings revealed a significant association between burnout and high levels of DP among 1st IPs who reported receiving inadequate support from their colleagues. Consistent with previous studies conducted among Irish junior physicians [11], physicians in Thailand [9, 18], and physicians in the United States [24, 25]. These studies emphasized the critical of peer support, individualized coaching, and teamwork in enhancing professional potential, managing stress, and improving career satisfaction. Incorporating social team-building activities into the work curriculum is strongly recommended [5, 37, 44]. Additionally, previous studies in Thailand shown a positive association between the lack of academic counselling services for intern physicians and reduced PA levels [18, 34]. These findings emphasize the importance of structured mentorship in mitigating burnout among intern physicians [45]. Mentorship is a crucial role in promoting fulfilment in medical practice by guiding trainees to navigate their training, achieve professional goals, and develop confidence in their abilities. Moreover, mentorship inspires physicians, enhances overall satisfaction, and encourages an understanding of enduring professional values and organizational culture while supporting skill and knowledge development within the workplace [44, 46]. Based on our findings, we recommend implementing a mentorship system, fostering a supportive buddy environment, and increasing remuneration. These measures are essential for reducing financial strain and promoting a healthier, more supportive work environment for intern physicians.

This study revealed several adverse effects associated with burnout among 1st IPs. Firstly, thoughts of resignation were strongly associated with EE, aligning with the findings of previous studies [18, 27, 47]. A previous study conducted in Japan found that dissatisfaction with income, poor work-life balance, and inadequate support from colleagues were significant factors associated with physicians’ intention to resign [48]. These findings suggest that such thoughts of resignation may be a direct consequence of burnout, underscoring the critical need for tailored support systems. Secondly, this study found that 1st IPs who perceived poor performance were linked to higher levels of DP and decreased PA, due to an intense and prolonged workload. This finding is consistent with previous studies [4, 9]. Chronic stress associated with burnout can impair cognitive processes, such as critical thinking, memory, and problem-solving abilities, which are essential for clinical judgment and performance [49]. Moreover, burnout may lead to a diminished sense of empathy or emotional connection with patients, an inadequate work-life balance, and insufficient support from family and friends [50]. Thirdly, high levels of DP were found to be positively associated with clinically harmful errors. This phenomenon is often linked to workplace factors such as excessive demands and inadequate resources. Although, burnout is not a clinical diagnosis, its occupational consequences are significant, as supported by previous findings [4, 5, 51]. Additionally, constant exposure to notifications can lower an individual’s threshold for addressing patient complaints, potentially impacting patient care [29]. Finally, no association was found between burnout and suicidal ideation among 1st IPs, indicating that burnout should be viewed as an occupational phenomenon distinct from depression. However, the strong association between depression and suicide remains significant [4]. Based on these findings, this study recommended the implementation of a comprehensive management system in hospitals to effectively mitigate burnout, reduce medical errors, and enhance the performance of intern physicians.

### Limitations

Our study had several limitations. Firstly, its cross-sectional design limits the ability to establish causal relationships between parameters and introduces temporal ambiguity, requiring caution in interpreting the observed association. Secondly, the use of social media for recruiting participants through an online survey may have introduced sampling bias, as individuals who are content or satisfied with their work conditions might have been less likely to participate. Thirdly, the limited participation of intern physicians resulted in a low response rate (15.3%), despite meeting the required statistical sample size. This low response rate may have led to selective bias, as individuals experiencing burnout or those particularly interested in the topic may have been overrepresented, potentially inflating the prevalence estimates. Fourthly, the variables chosen for assessing working conditions and work performance were based on the authors’ own selection rather than a validated questionnaire, may introduce subjectivity and limit the generalizability of the findings. Finally, the reliance on self-reported data may have resulted in recall bias, particularly considering that most of the data collection occurred during the final two months of the participants’ internships.

## Conclusions

In this study, 1st IPs experienced a higher prevalence of burnout during first-year internships. Those who lacked adequate support from colleagues and access to academic counselling services, faced income-workload imbalances, and experienced professional dissatisfaction were at a significantly higher risk of burnout. High levels of burnout also led to poorer performance and increased thought of resignation. To address these challenges, interventions such as mental health surveillance, EAP, increased pay, mentorship program, buddy support systems, and adherence to work-hour guidelines should be implemented. Further research is needed to establish a cause-and-effect relationship between burnout and its contributory to this population, ideally through a longitudinal study.

## Electronic supplementary material

Below is the link to the electronic supplementary material.

Supplementary Material 1:Additional File 1 of Physician burnout, associated factors, and their negative consequences on work performance during the first-year internship in Thailand: A cross-sectional study Additional file 1: Supplementary file 1. Questionnaire Items of Burnout, Demographic and Health Characteristics, Working Conditions, and Work Performance of Intern Physicians During First-Year Internship. Supplementary file 2. The province-by-province distribution of 412 intern physicians responding to a survey in Thailand through an online platform from all regions of Thailand, 2022. The figure was created using Bing.©GeoNames, Microsoft, Navinfo, OpenStreetMap, TomTom. Supplementary file 3. Estimated-Scores of intern physicians responding to a survey in Thailand through an online platform to Burnout Questions of Each Subscale by Forest Plot.

## Data Availability

The dataset supporting the conclusions of this article is used and/or analyzed during the current study are available from the corresponding author (PS) on reasonable request.

## Abbreviations

ACGME: Accreditation Council for Graduate Medical Education
Aor: Adjusted odds ratio
CI: Confidence interval
DP: Depersonalization
EAP: Employee assistance program
EE: Emotional exhaustion
ICD: International classification of diseases
PA: Personal accomplishment
SD: Standard deviation
1^st^: IPs First-year intern physicians
