# Supplementary material for: Physician burnout, associated factors, and their effects on work performance throughout first-year internships during the COVID-19 pandemic in Thailand: a cross-sectional study
Source: BMC Public Health. 2025 May 28;25:1967. doi: 10.1186/s12889-025-23172-7 (PMC12117941; doi:10.1186/s12889-025-23172-7)
Supplement: Supplementary file 1 — Supplementary Material 1:Additional File 1 of Physician burnout, associated factors, and their negative consequences on work performance during the first-year internship in Thailand: A cross-sectional study Additional file 1: Supplementary file 1. Questionnaire Items of Burnout, Demographic and Health Characteristics, Working Conditions, and Work Performance of Intern Physicians During First-Year Internship. Supplementary file 2. The province-by-province distribution of 412 intern physicians responding to a survey in Thailand through an online platform from all regions of Thailand, 2022. The figure was created using Bing.©GeoNames, Microsoft, Navinfo, OpenStreetMap, TomTom. Supplementary file 3. Estimated-Scores of intern physicians responding to a survey in Thailand through an online platform to Burnout Questions of Each Subscale by Forest Plot. [file 12889_2025_23172_MOESM1_ESM.docx]

Supplementary File 1

Questionnaire Items of Burnout, Demographic and Health Characteristics, Working Conditions, and Work Performance of Intern Physicians During First-Year Internship**.**

Are you a first-year Thai intern physician with at least 6 months of training in 2021 academic year?

[ ] Yes [ ] No

**Section 1: General personal information**

1. Gender [ ] Male [ ] Female

2. Age _____ years old

3. Physical underlying diseases (such as allergic rhinitis, migraine, myofascial pain, etc.)

[ ] None

[ ] Present: name of the diseases _______________________

4. Current medication

[ ] None

5. Marriage status

[ ] Single [ ] Married

[ ] Divorced/Widowed [ ] Separated

6. Please estimate the average net salary per month, including overtime and extra income from medical-related jobs, ______________ THB per month

**Section 2: Occupation-related factors**

1. What is your current work hour on average?

1.1 Office hours: _____________ hours per week

1.2 Duty and overtime: _____________ hours per week

2. How frequently have your colleagues supported you?

[ ] None

[ ] Sometimes

[ ] Most of the time

3. How frequently have your colleagues provided academic counseling at the workplace?

[ ] None

[ ] Sometimes

[ ] Most of the time

4. What is your perception of the current amount of work to financial compensation, balance income, and workload?

[ ] Fair

[ ] Unfair

5. What is your satisfaction with the medical profession?

[ ] Satisfied

[ ] Neutral

[ ] Dissatisfied

6. Have you ever thought of resigning from your current work?

[ ] No

[ ] Yes, please describe the reasons ___________________

**Section 3: Burnout assessment**

| **Statements** | Never | 2-3 times a year | Once a month | 2-3 times a month | Once a week | 2-3 times a week | Every day |
| --- | --- | --- | --- | --- | --- | --- | --- |
| I feel emotionally exhausted because of my work |  |  |  |  |  |  |  |
| I feel worn out at the end of a working day |  |  |  |  |  |  |  |
| I feel tired as soon as I get up in the morning and see a new working day stretched out in front of me |  |  |  |  |  |  |  |
| I can easily understand the actions of my colleagues/supervisors |  |  |  |  |  |  |  |
| I get the feeling that I treat some clients/colleagues impersonally, as if they were objects |  |  |  |  |  |  |  |
| Working with people the whole day is stressful for me |  |  |  |  |  |  |  |
| I deal with other people’s problems successfully |  |  |  |  |  |  |  |
| I feel burned out because of my work |  |  |  |  |  |  |  |
| I feel that I influence other people positively through my work |  |  |  |  |  |  |  |
| I have become more callous to people since I have started doing this job |  |  |  |  |  |  |  |
| I’m afraid that my work makes me emotionally harder |  |  |  |  |  |  |  |
| I feel full of energy |  |  |  |  |  |  |  |
| I feel frustrated by my work |  |  |  |  |  |  |  |
| I get the feeling that I work too hard |  |  |  |  |  |  |  |
| I’m not really interested in what is going on with many of my colleagues |  |  |  |  |  |  |  |
| Being in direct contact with people at work is too stressful |  |  |  |  |  |  |  |
| I find it easy to build a relaxed atmosphere in my working environment |  |  |  |  |  |  |  |
| I feel stimulated when I have been working closely with my colleagues |  |  |  |  |  |  |  |
| I have achieved many rewarding objectives in my work |  |  |  |  |  |  |  |
| I feel as if I’m at my wit’s end |  |  |  |  |  |  |  |
| In my work, I am very relaxed when dealing with emotional problems |  |  |  |  |  |  |  |
| I have the feeling that my colleagues blame me for some of their problems |  |  |  |  |  |  |  |

**Section 4: Psychological problems**

1. Recently, you have been feeling unhappy and depressed.

| Strongly disagree | Not agreed | Neutral | Agreed | Strongly agree |
| --- | --- | --- | --- | --- |

2. In the last 1 month, on average, what is your sleep hours per day, ____________ hours/day

3. Do you have any suicidal ideation in the past 12 months?

[ ] No

[ ] Yes

4. Please evaluate your work performance during the last 12 months. Answer…………

0 1 2 3 4 5 6 7 8 9 10

Poor work performance

Maximum work performance

**Section 5: Impact on medical services**

What is the latest time that you experienced the error during your first-year internship?

|  | Yes | Never |
| --- | --- | --- |
| 1. I have been involved in a major medical error that was harmful to the patient. |  |  |
| 2. I have been doing a minor medical error. |  |  |
| 3. Medication error |  |  |
| 4. Laboratory and investigation error |  |  |

Supplementary file 2

The province-by-province distribution of 412 intern physicians responding to a survey in Thailand through an online platform from all regions of Thailand, 2022. The figure was created by Bing.©GeoNames, Microsoft, Navinfo, OpenStreetMap, TomTom.


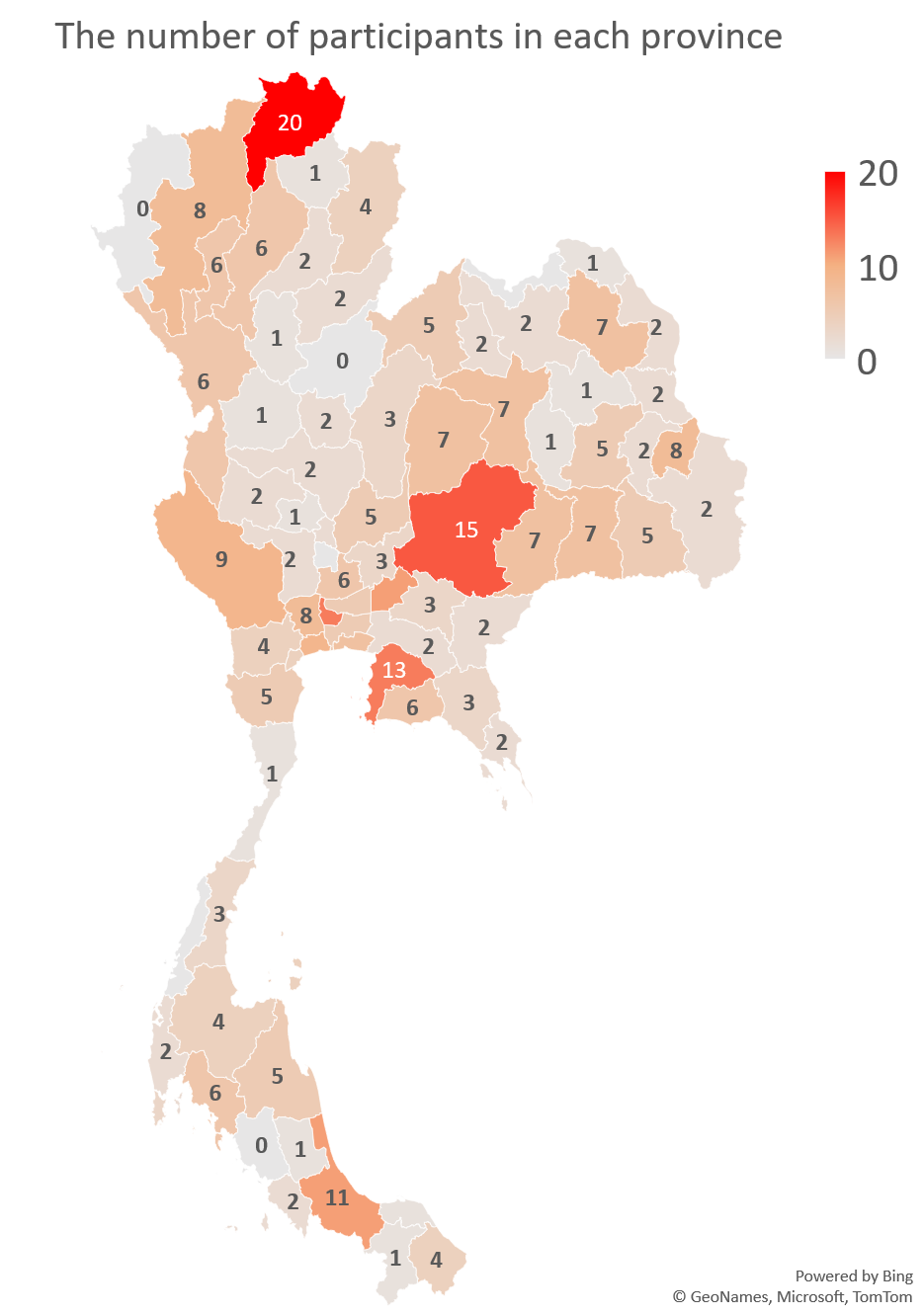


Supplementary file 3


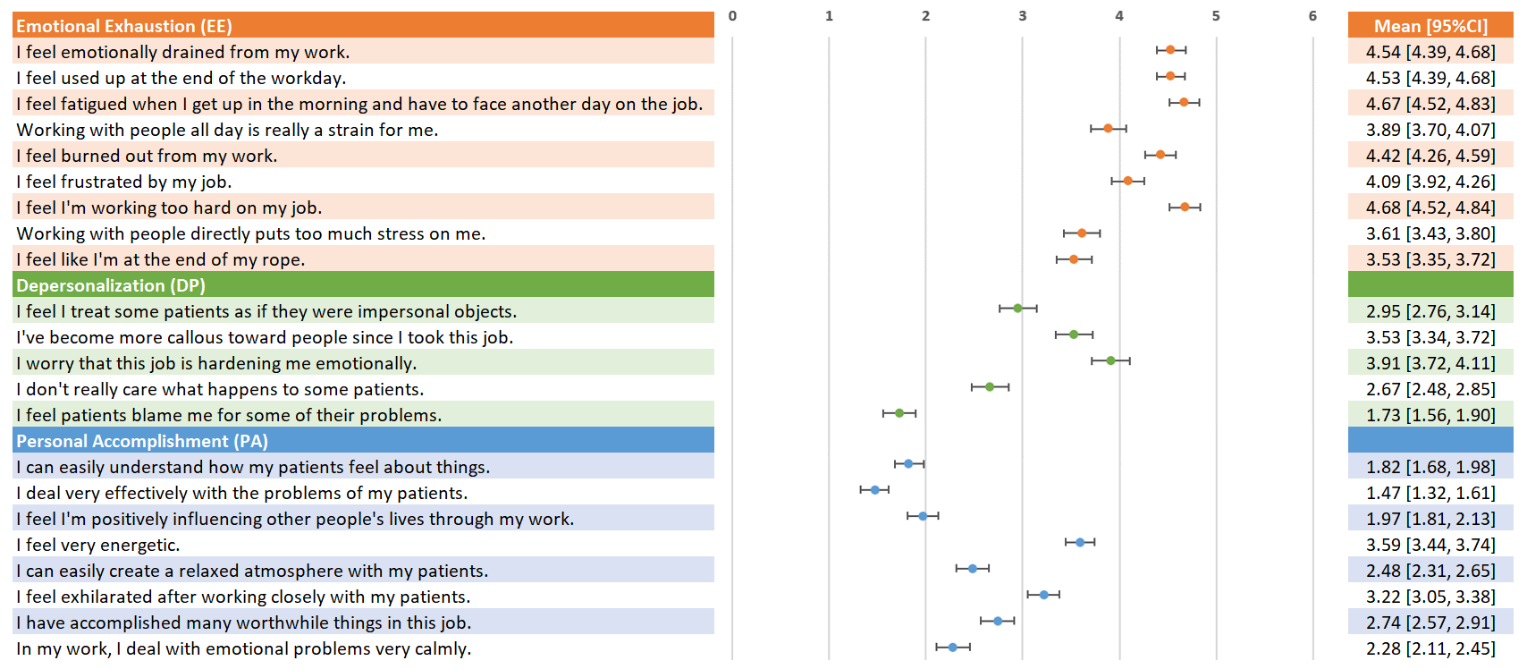
Estimate-Scores of intern physicians responding to a survey in Thailand through an online platform to Burnout Questions of Each Subscale by Forest Plot.
